# Neutrophil elastase as a potential biomarker related to the prognosis of gastric cancer and immune cell infiltration in the tumor immune microenvironment

**DOI:** 10.1038/s41598-023-39404-y

**Published:** 2023-08-18

**Authors:** Wangqiang Jia, Qianwen Luo, Jiang Wu, Yuanchao Shi, Quanlin Guan

**Affiliations:** 1 The First Clinical Medical College of Lanzhou University, Lanzhou, China; 2 Department of Oncology Surgery, The First Hospital of Lanzhou University, No. 1, Donggang West Road, Lanzhou, 730000 Gansu Province China

**Keywords:** Cancer, Biomarkers, Oncology

## Abstract

Exploring biomarkers interrelated the tumor immune microenvironment (TIME) provides novel ideas for predicting the prognosis of gastric cancer (GC) and developing new treatment strategies. We analyzed the differential gene expression levels between the high and low StromalScore and ImmuneScore groups. Neutrophil elastase (ELANE) was evaluated as a potential biomarker by conducting intersection analysis of the protein–protein interaction network and univariate Cox regression analysis. The expression of ELANE was evaluated by immunohistochemistry. Its prognostic value was evaluated using Kaplan–Meier (K–M) survival curves and multivariate Cox regression analysis and its potential biological molecular mechanism was examined by gene set enrichment analysis (GSEA). We applied the CIBERSORT computing method to analyze the relationship between ELANE and tumor immune-infiltrating cells (TIICs). K–M survival curve showed that higher ELANE expression was closely related to shorter overall survival. The Cox regression analysis indicated that the high expression of ELANE was an independent prognostic risk factor in patients with GC. The GSEA revealed that genes in the ELANE high-expression group were involved in the signaling pathways regulating immune response; genes in the ELANE low-expression group were involved in the signaling pathways that regulate metabolism. ELANE might be participate in the change of TIME from immunodominant to metabolically dominant and its expression was closely related to tumor mutation burden and multiple TIICs. ELANE is a potential biomarker for predicting the GC patients’ survival and prognosis. It influences the tumor immune cell infiltration in the TIME, and affects the TIME to maintain their immune status.

## Introduction

According to the Global Cancer Statistics in 2020, Gastric cancer (GC) ranks fifth (5.6%) among common malignant tumors and fourth (7.7%) among cancer related deaths^1^. In China, the latest cancer statistics in 2022 reported that GC ranked third in terms of incidence and mortality^2^. Patients with GC do not usually have obvious symptoms at the beginning of the disease, and most of the patients had already reached the advanced stage when diagnosed^3^. Advanced-stage GC is characterized by poor prognosis with limited treatment strategies that are applicable. At present, immunotherapy is one of the primary treatment methods for advanced-stage GC. However, only a few patients can benefit from immunotherapy. Therefore, potential biomarkers that can accurately predict the prognosis and serve as the target of immunotherapy for GC should be determined. Several studies showed that the changes in TIME had important clinical significance in predicting the tumor prognosis and therapeutic effect^4^. The changes of the TIME can promote tumor growth and inhibit the tumor’s response to antitumor therapy^5^. Therefore, exploring biomarkers related to the TIME will provide novel ideas for predicting GC patients’ prognosis and developing new therapeutic strategies.

Stromal score is an indicator used to evaluate the composition and structure of extracellular stromal around tumors, while immune score is an indicator used to evaluate the degree and type of immune cell infiltration around tumors. TIME refers to the sum of immune cells, cytokines, and other immune related components around the tumor. An increasing amount of evidence has suggested the clinical importance of stromal cells and immune cells in the microenvironment of GC tissues^6^. Stromal score and immune score can reflect the state and characteristics of TIME, which is of great significance for predicting the development and prognosis of tumors. Studies have shown that stromal scores and immune scores are closely related to the immunotherapeutic efficacy of tumors and the survival rate of GC patients^7,8^. The ESTIMATE algorithm can predict the purity of our tumors by predicting immune scores and stromal scores, thereby predicting the content of stromal and immune cells. If the content of stromal cells and immune cells is high, then the purity of the tumor is low. If the content of stromal cells and immune cells is reduced, the purity of the tumor will be higher^9^. In order to better understand the impact of immune and stromal cell related genes on prognosis, we used ESTIMATE and CIBERSORT computational methods to calculate the TIICs proportion and the ratio of immune and stromal components of GC samples from The Cancer Genome Atlas (TCGA) database.

In this study, multiple analysis methods were used with TCGA data to ultimately identify Neutrophil elastase (ELANE), and the actual IHC expression of ELANE and functional analysis were conducted through GSEA analysis. Through this analysis, we aim to discover new prognostic biomarkers related to the TIME of stroma and tumor in GC and elucidate their biological functions.

## Materials and methods

### Data collection

In this study, we used the standardized RNA sequencing dataset of GC in The Cancer Genome Atlas (TCGA) database (https://portal.gdc.cancer.gov), and downloaded complete clinical parameters, such as gender, age, grade, clinical stage, TNM, survival time, and survival status. This study included 375 GC samples and 32 normal samples. We collected the paraffin blocks of tumor (86 cases) and normal tissue (20 cases) specimens from the Department of Oncology Surgery of the First Hospital of Lanzhou University from January 2018 to December 2018. TCGA datasets were used as the training cohort, and clinical samples were used as the validation cohort.

### Evaluation of ImmuneScore, StromalScore, and ESTIMATEScore

ESTIMATE^9^, a method that uses gene expression signatures to infer the fractions of stromal and immune cells in tumor samples, was used to evaluate the levels of immune cell infiltration (immune score), the stromal content (stromal score), the stromal-immune comprehensive score (ESTIMATE score) and tumor purity for each GC sample. We used the “estimate” package of R language^10^ to calculate the proportion of stromal and immune components of each tumor sample, and the results were expressed as ImmuneScore, StromalScore, and ESTIMATEScore. Based on the median ImmuneScore and StromalScore of each tumor sample, we divided all samples into high and low groups.

### Analysis of differentially expressed genes between the high and low ImmuneScore and StromalScore groups

We applied the “edgeR” package of R language to analyze the differentially expressed genes (DEGs) between the high and low ImmuneScore and StromalScore groups. The genes with false discovery rate (FDR) < 0.05 and | logFC (FoldChange) |> 1 were considered as DEGs. We applied the “pheatmap” package of R language to draw a heatmap to visualize the expression of DEGs in GC tissues^10^. A Venn diagram was used to analyze the intersection of DEGs between the high and low ImmuneScore and StromalScore groups. The number of shared DEGs that were upregulated and downregulated in these groups were obtained.

### Establishment of protein–protein interaction network

The PPI network for the screened DEGs was constructed by the STRING database (https://string-db.org), and Python refactoring version 3.9.0 was subsequently used for visualization. The core nodes in the PPI network were obtained by sorting based on the number of nodes, and a bar chart was constructed for visualization.

### Establish enrichment analysis

We applied the “clusterProfiler,” “enrichment plot,” and “ggplot2” packages of R language to conduct Gene Ontology (GO) function enrichment analysis and Kyoto Encyclopedia of Genes and Genomes (KEGG) biological pathway analysis of DEGs^11,12^. A *p*-value of < 0.05 indicated statistical significance. We download the C2 gene set (C2. cp.kegg. v2022.1. Hs. symbols) from the database and served it as the target gene set for gene set enrichment analysis (GSEA). GSEA of target genes was conducted by the GSEA software version 4.3.2. |NES |> 1, NOMp < 0.05 and FDRq < 0.05 were considered as the criteria for significant enrichment.

### Cox regression receiver operating characteristic curve analysis

Used univariate Cox regression analysis to analyze the patients’ prognosis with DEGs shared by ImmuneScore and StromalScore by the “survival” package in R language^13^. The most significant factors were obtained according to the order of *p*-values (from small to large). Then, a Venn map was used to perform an intersection analysis between the most significant factors obtained in the univariate Cox regression analysis and the core nodes in the PPI network to screen out the target genes. We applied the “survival” package of R language to analyze the survival of GC patients and draw a K–M curve. A *p*-value of < 0.05 indicated statistical significance. ROC curve was drawn using the “survivalROC” package of R software to assess the clinical value of target genes in predicting the survival^14^. An area under the curve (AUC) of > 0.5 was considered significant. At the same time, we applied multivariate Cox regression analyses to explore the risk factors of GC patients’ prognosis. A *p*-value of < 0.05 indicated statistical significance.

### Analysis of TIICs and immune checkpoint genes

Used the “CIBERSORT” package^15^ of R language to quantitatively analyze the infiltration abundance of immune cells in the TIME and screen out the immune cells related to the target genes. Meanwhile, the Spearman’s correlation was used to explore the relationship between target genes and TIICs. In addition, the relationship between target gene expression and immune checkpoint (IC) genes was analyzed; a negative correlation coefficient indicated a negative correlation, while a positive correlation coefficient indicated a positive correlation. A *p*-value of < 0.05 indicated statistical significance.

### Immunohistochemistry

Immunohistochemistry (IHC) were used to detect the expression of ELANE protein in gastric cancer tissue and adjacent normal tissues. After deparaffinization and hydration, the sections were subjected to antigen retrieval in 20 × Tris EDTA antigen repair solution (pH 9.0), and then incubated overnight with anti ELANE antibodies (1:200, Bios, China, product number bs-23548R) at 4 °C. After incubating with the secondary antibody (HRP labeled goat anti rabbit IgG), used 3.3′-Diaminobenzidine (DAB) to stain the sections, and then incubated with Haematoxylin. The sections were analyzed with an optical microscope.

H-Score is a method for evaluating immunohistochemical staining results, mainly used to evaluate protein expression levels. H-SCORE = ∑ (pi × i) = (percentage of weak intensity × 1) + (percentage of moderate intensity × 2) + (percentage of strong intensity × 3). The H-Score range from 0 to 300, with higher scores indicating higher expression levels^16,17^.

### Ethics approval and consent to participate

As this study used an open database, there was no requirement for ethical approval and consent to participate.

## Results

### DEGs screened by ImmuneScore and StromalScore in the TIME

**Figure 1:**
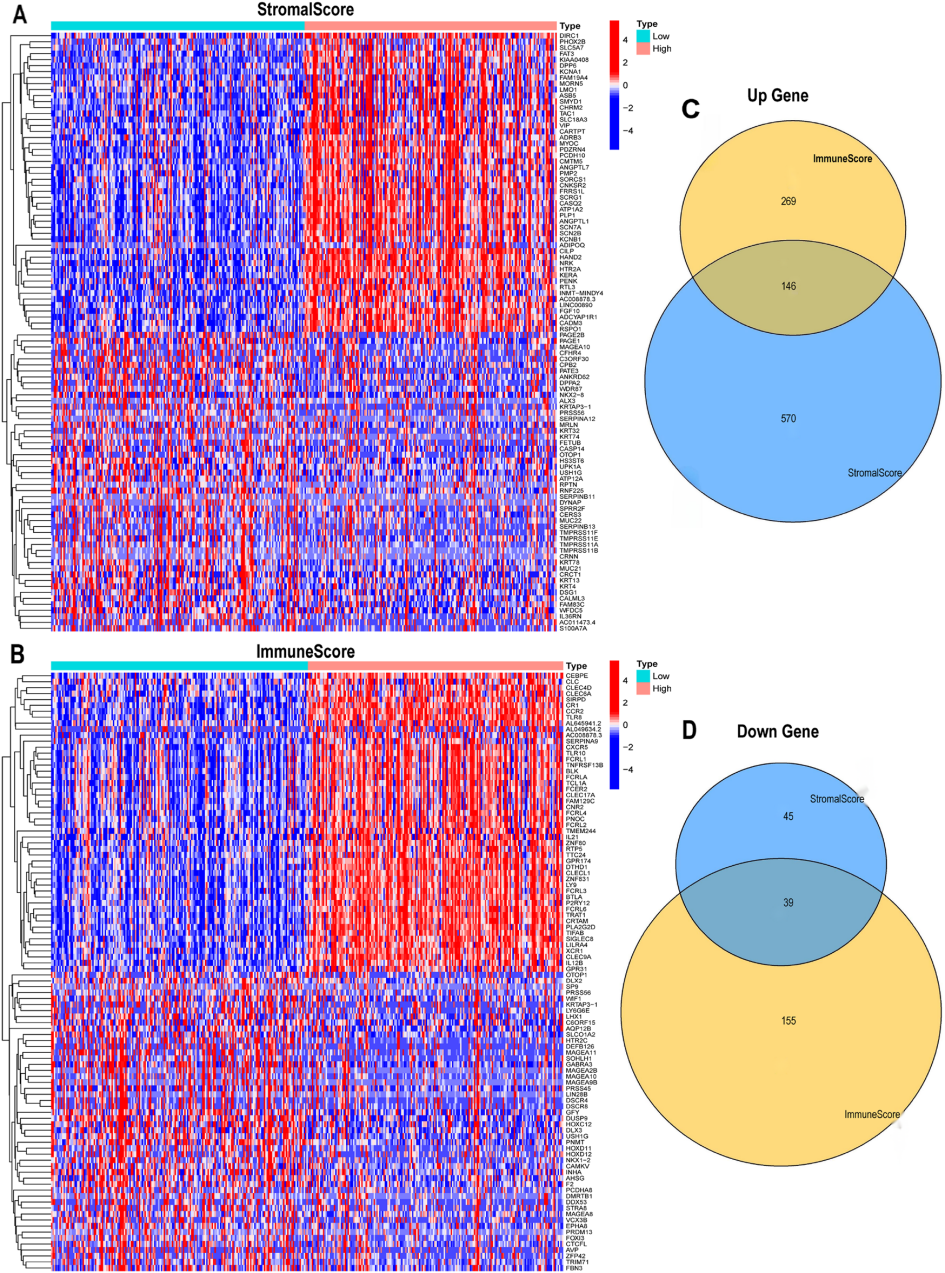
Screening of differentially expressed genes (DEGs). (**A**, **B**) Heatmaps of top 100 DEGs according to stromal scores and immune scores. (**C**, **D**) The number of shared upregulated and downregulated genes by ImmuneScores and StromalScores.

375 GC samples were divided into high-score and low-score groups based on the median of ImmuneScore and StromalScore. To explore the changes of the immune and stromal components in the TIME, we performed a series of comparative analyses of GC samples with high and low scores. | logFC|> 1 was used as the screening criteria, and a *p*-value of < 0.05 indicated statistical significance. In the StromalScore group, 800 DEGs were obtained, of which 716 were upregulated genes and 84 were downregulated genes. 609 DEGs were identified in the ImmuneScore group, of which 415 were upregulated genes and 194 were downregulated genes. Finally, 1409 DEGs were obtained, of which 1131 were downregulated genes and 278 were upregulated genes. The expression of differential genes was visualized using heatmaps (Fig. 1A,B). The Venn diagram showed that 146 up-regulated genes (Fig. 1C) and 39 down-regulated genes (Fig. 1D) were shared by the ImmuneScore and StromalScore groups. Finally, 185 DEGs were finally obtained through the analysis of Venn diagram.

### GO and KEGG enrichment analyses results of DEGs

**Figure 2:**
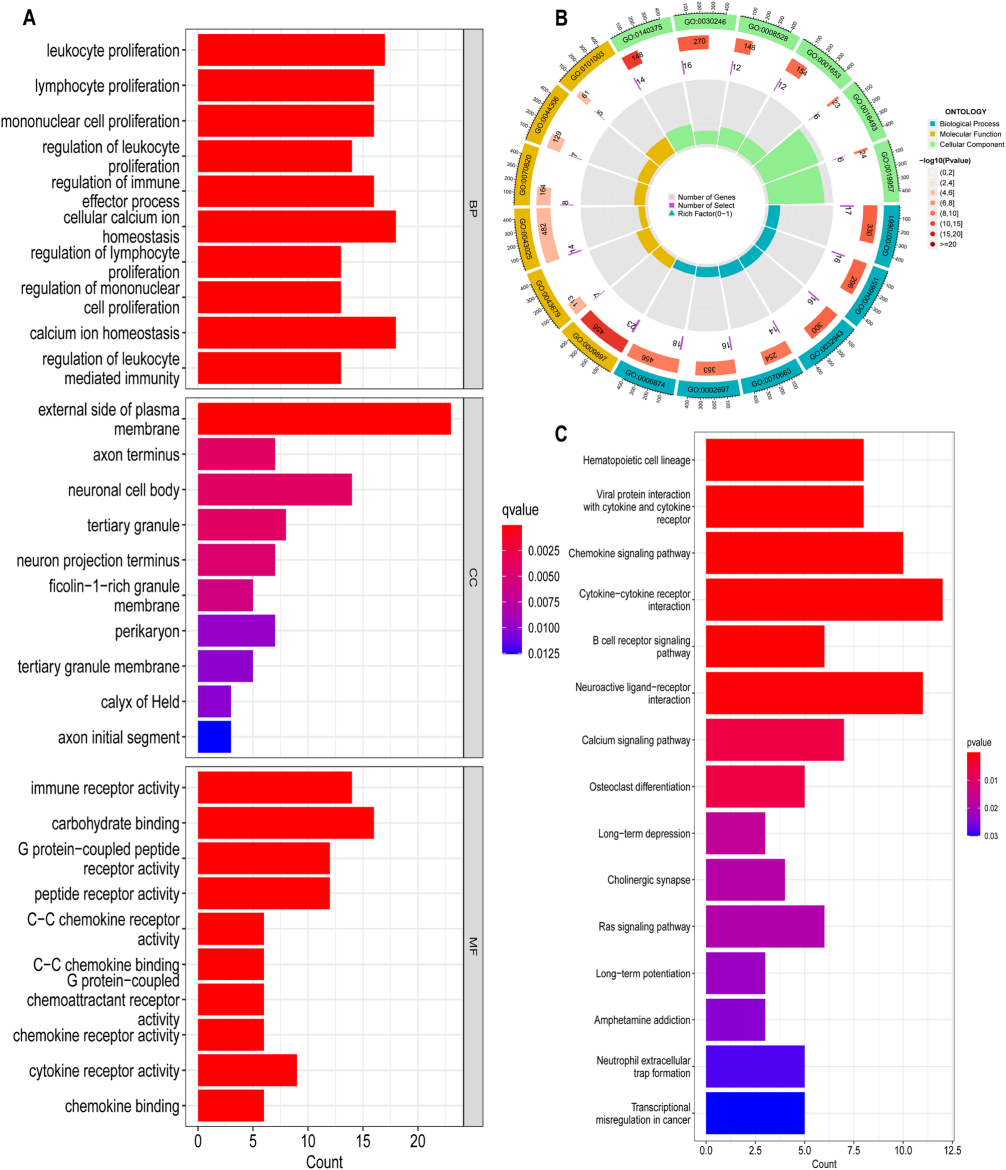
Functional enrichment analysis of 185 DEGs. (**A**, **B**) GO enrichment analysis of 185 DEGs. (**C**) KEGG enrichment analysis of 185 DEGs.

In order to understand the molecular mechanism the above 185 DEGs affecting the TIME of GC patients, GO and KEGG functional analyses were conducted. The GO results indicated that these DEGs primarily participated in the following biological processes: leukocyte proliferation and regulation, lymphocyte proliferation and regulation, monocyte proliferation and regulation, regulation of immune effect process, and leukocyte mediated immune regulation. In terms of the GO molecular function, DEGs mainly focused on regulating the immune receptor activity, cytokine receptor activity, chemokine receptor activity, chemokine binding, etc. (Fig. 2A,B). KEGG results indicated that these DEGs primarily participated in the cytokine-cytokine receptor interactions, chemokine signaling pathways, viral protein-cytokine and cytokine receptor interactions, and B-cell receptor signaling pathways (Fig. 2C). These DEGs might be correlated with the regulation of immune response and TIME in patients with GC.

### Interactive analysis results of PPI network and univariate Cox regression analysis

**Figure 3:**
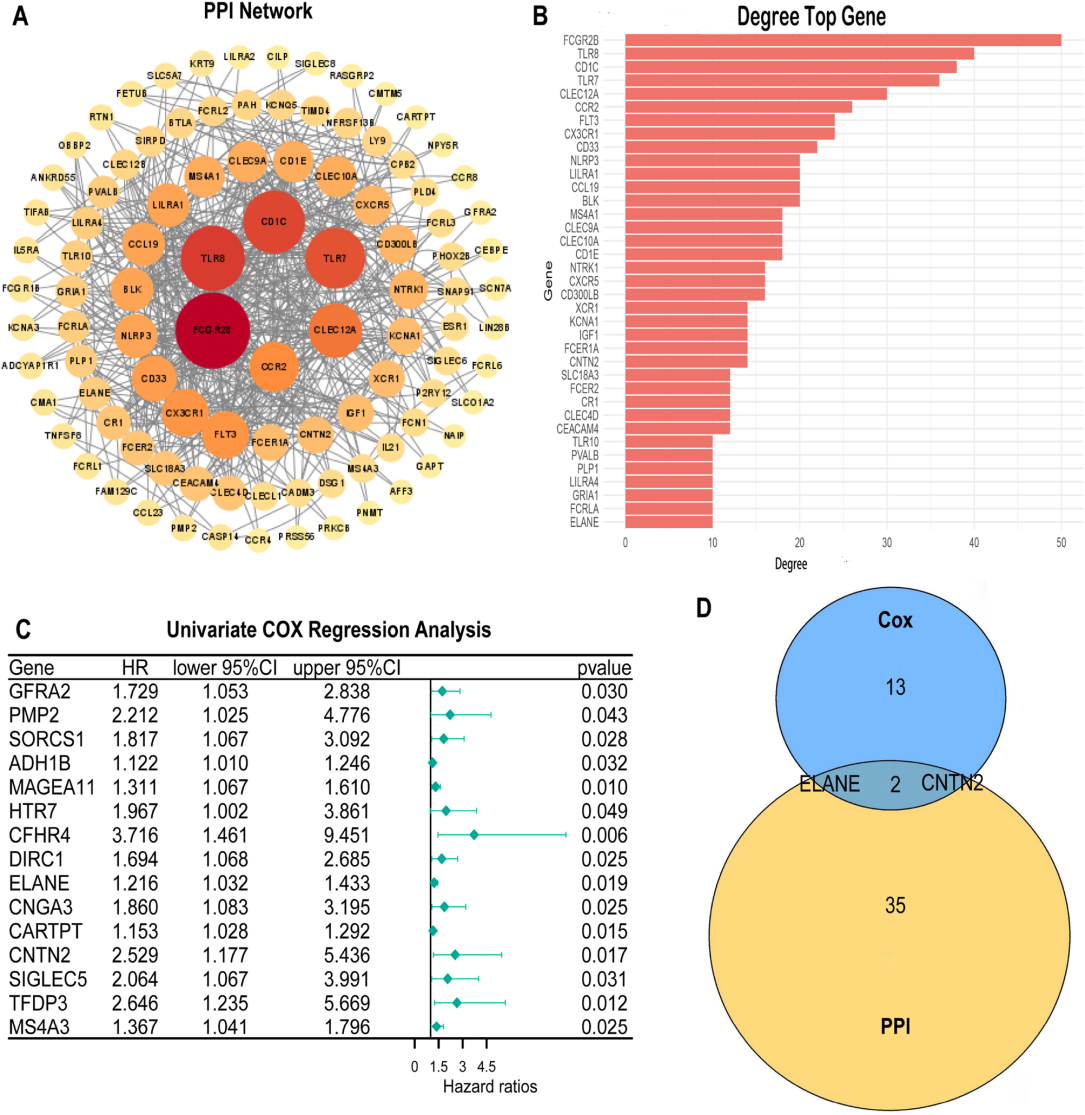
PPI network and univariate Cox regression analysis of DEGs. (**A**) PPI network of DEGs. (**B**) Top 37 DEGs according to the number of degrees. (**C**) Univariate Cox regression analysis of DEGs, listing the top significant factors with a *p*-value of < 0.05. (**D**) Venn diagram showing the genes shared by top significant factors in the univariate Cox regression analysis and PPI network.

To further determine the interaction among the 185 DEGs, a PPI network was drawn (Fig. 3A) and the top 37 genes were screened according to the number of nodes (Fig. 3B). To explore the impact of 185 DEGs on the GC patients’ survival, univariate Cox regression analysis was conducted to screen out the 15 genes with the most significant impact (Fig. 3C). Finally, results of the intersection analysis between the leading nodes of the PPI network and 15 genes screened by univariate Cox regression revealed that only two genes, ELANE and CNTN2, overlapped (Fig. 3D). In this study, we selected ELANE for subsequent analyses.

### Correlation between ELANE expression level and the clinical parameters of GC patients

**Figure 4:**
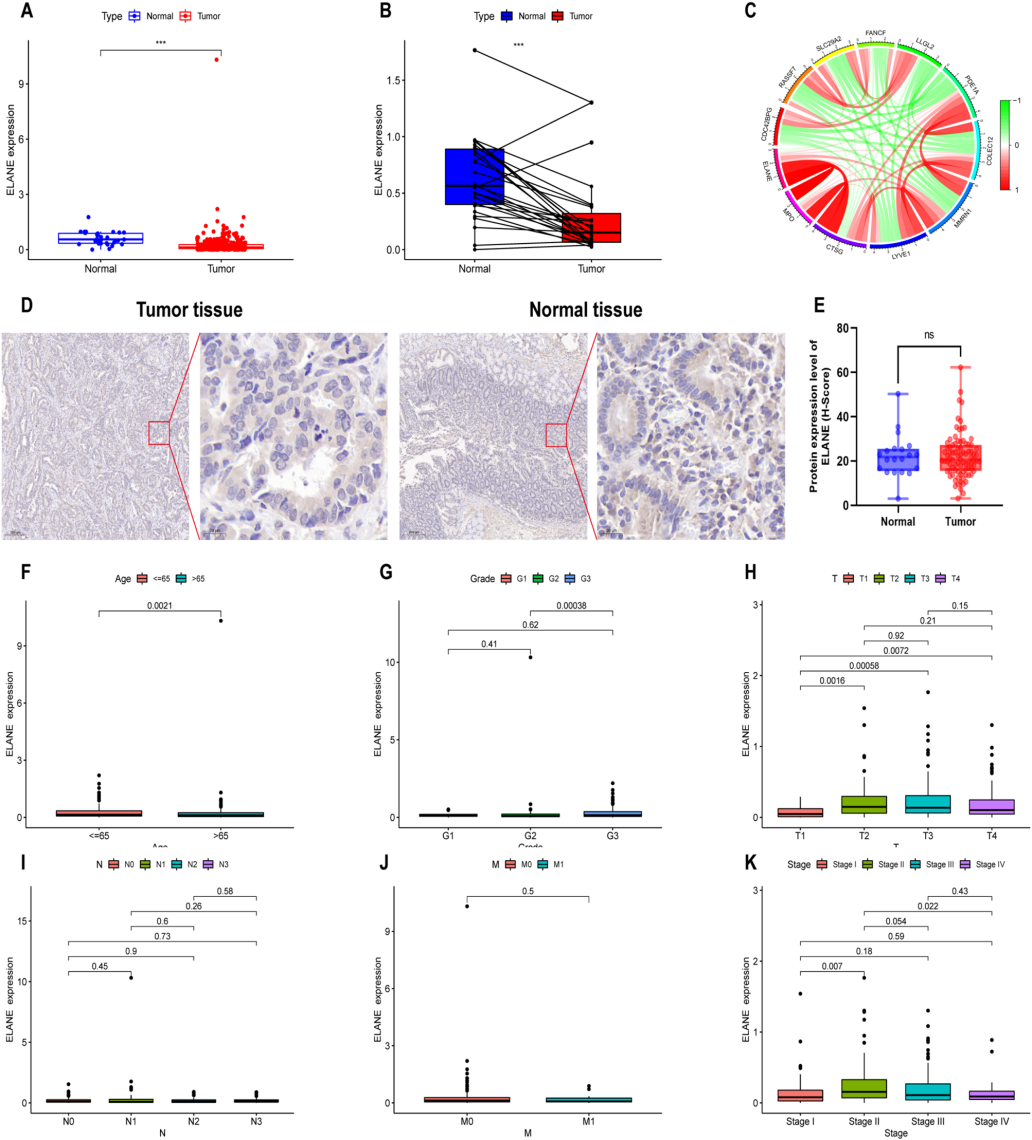
Expression level of neutrophil elastase (ELANE) and its relationship with clinical parameters in patients with gastric cancer (GC). (**A**) Differentiated expression of ELANE in the normal and tumor samples. (**B**) Paired differentiation analysis of the expression level of ELANE in the normal and tumor samples derived from the same patient. (**C**) Genes co-expressed with ELANE. (**D**–**E**) Immunohistochemistry analysis of ELANE protein expression in GC and normal tissues. (**F**–**K**) Relationship between the expression level of ELANE and clinical parameters of patients with GC. *p < 0.05, **p < 0.01, ***p < 0.001, ****p < 0.0001 here and in the following figures.

The mRNA expression of ELANE was analyzed based on the TCGA database. The expression of ELANE was low in GC samples and high in normal samples (*p* < 0.001, Fig. 4A). When the expression level of ELANE in tumor and normal tissues of the same GC patient was compared, the expression of ELANE in normal tissues was also obviously higher than that in tumor (*p* < 0.001, Fig. 4B). As shown in Fig. 4C, ELANE was mainly co-expressed with Myeloperoxidase (MPO), Cathepsin G (CTSG), Multimerin-1 (MMRN1), Collectin-12 (COLEC12) and Phosphodiesterase 1A (PDE1A). However, using IHC staining for 86 pairs of tumor and normal tissues, there was no difference in ELANE protein expression in GC tissues compared to normal tissues (*p* > 0.05, Fig. 4D,E). Meanwhile, immunohistochemistry results showed that the ELANE protein was localized in the cytoplasm (Fig. 4D). Analysis of the ELANE expression level and clinical parameters of GC patients indicated that the ELANE expression was related to age (*p* = 0.0021, Fig. 4F). The expression of ELANE in T1 stage was obviously different from T2, T3, and T4 stages (*p* < 0.05, all), and the expression level of T1 stage was lower than T2, T3, and T4 stages (Fig. 4H). However, no difference was found in the ELANE expression levels among T2, T3, and T4 stage patients (*p* > 0.05) (Fig. 4H). Similarly, the ELANE expression in stage I was significantly different from stage II (*p* < 0.05), while the ELANE expression level of stage I was lower than stage II, III, and IV; however, no difference was found in the ELANE expression level between stage II, III, and IV (*p* > 0.05, Fig. 4K). In addition, no correlation was observed between ELANE expression levels and tumor grade, N stage, or M stage (*p* > 0.05, Fig. 4G–J). The above results revealed that the expression of ELANE showed an upregulated trend with the progression of GC, which indicated that the high expression of ELANE might be closely linked to tumor progression in GC patients.

### The predictive value of ELANE in survival and prognosis of patients with GC

**Figure 5:**
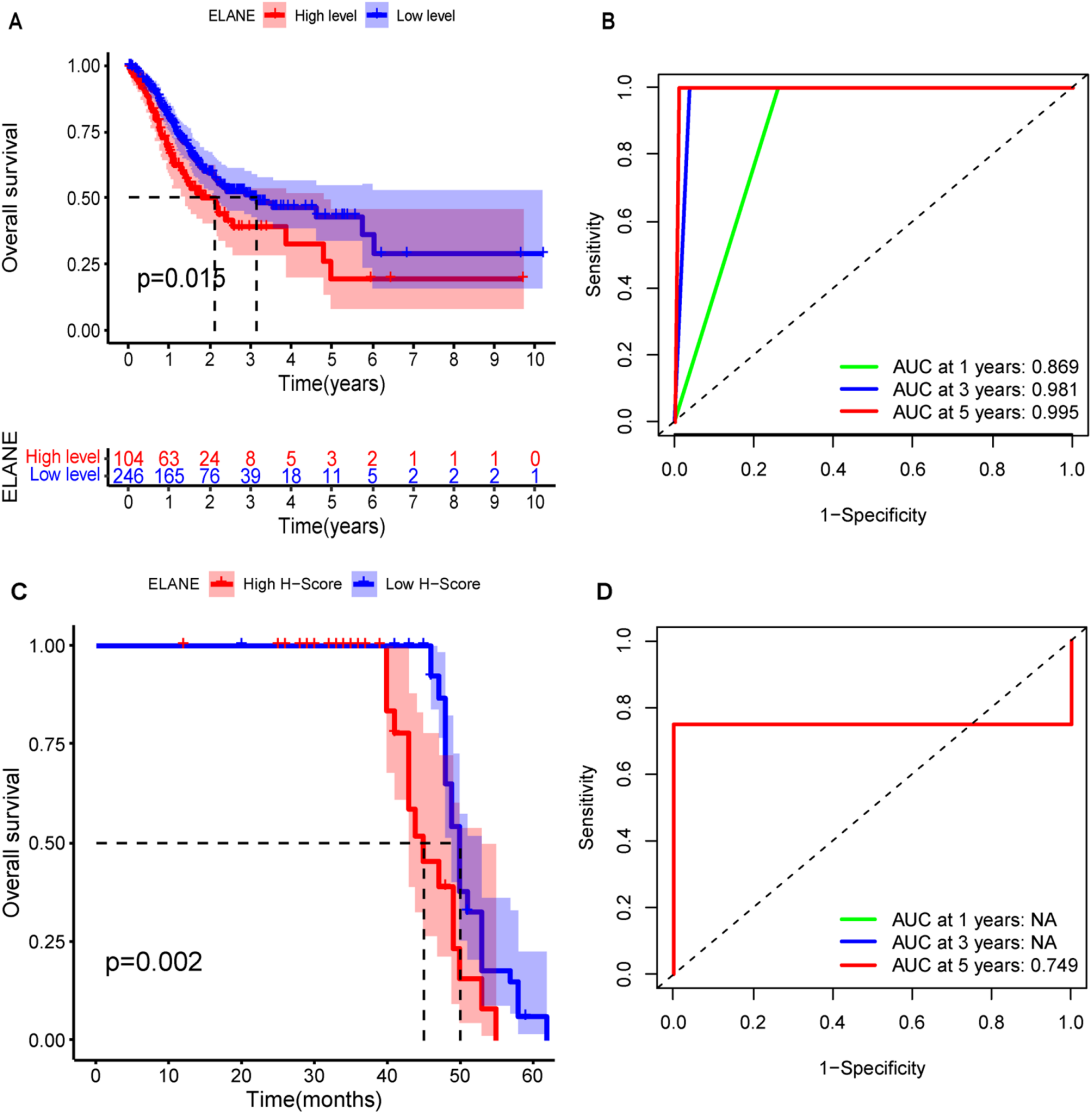
K–M curve and ROC of GC patients based on the level of neutrophil elastase (ELANE) expression. (**A**) Survival analysis of GC patients with high and low ELANE expression in TCGA database. (**B**) Predictive value of ELANE on the 1-year, 3-year, and 5-year survival periods of GC patients in TCGA database. (**C**) K–M curve for overall survival in high and low ELANE expression group based on validation cohort. (**D**) Predictive value of ELANE on the 1-year, 3-year, and 5-year survival periods of GC patients in validation cohort.

In this study, all samples were divided into ELANE high- and low-expression groups, and K–M survival curve was conducted to determine the OS of all patients. In TCGA cohort, the OS of GC patients with low ELANE expression level was longer than those with high ELANE expression level (*p* = 0.015, Fig. 5A), and the *p*-value was less than 0.05. In validation cohort, we observed identical results (*p* = 0.002, Fig. 5C). In order to test the predictive value of ELANE expression level on the survival cycle of GC patients, a ROC was established in this study. In TCGA cohort, ROC indicated that the 1-year, 3-year, and 5-year AUC values for GC patients were 0.869, 0.981, and 0.995, respectively (Fig. 5B). In validation cohort, ROC indicated that the 5-year AUC value for GC patients was 0.749 (Fig. 5D). Therefore, ELANE was considered as a good predictor of survival. Meanwhile, Cox regression analysis was also carried out. The univariate Cox regression analysis results showed that age (*p* = 0.003), T (*p* = 0.026), N (*p* = 0.006), M (*p* = 0.003), high StromalScore (*p* = 0.046), and high expression level of ELANE (*p* = 0.009) were risk factors for the prognosis of GC patients (Table 1). However, multivariate Cox regression analysis showed that age (*p* < 0.001), N (*p* = 0.03), M (*p* < 0.001), and high ELANE expression level (*p* = 0.019) were independent risk factors for the prognosis of GC (Table 1). Therefore, the expression of ELANE was negatively related to the GC patients’ survival cycle; moreover, ELANE is a potential biomarker that can predict the GC patients’ survival and prognosis.

**Table 1:** Results of the univariate and multivariate Cox regression analyses based on TCGA database.

Variables	Univariate analysis		Multivariate analysis	
	Hazard ratio (95%CI)	*P* value	Hazard ratio (95%CI)	*p* value
Age(y ≥ 55/< 55)	1.028 (1.009–1.047)	0.003	1.045 (1.024–1.066)	< 0.001
Gender	1.579 (1.041–2.398)	0.032	1.635 (1.069–2.499)	0.023
Grade	1.349 (0.938–1.940)	0.106	1.388 (0.924–2.087)	0.115
T	1.300 (1.031–1.639)	0.026	1.253 (0.957–1.641)	0.101
M	2.456 (1.345–4.485)	0.003	3.301 (1.734–6.286)	< 0.001
N	1.268 (1.071–1.501)	0.006	1.224 (1.019–1.471)	0.030
StromalScore	1.000 (0.999–1.000)	0.046	1.000 (0.998–1.001)	0.480
ImmuneScore	0.998 (0.997–1.000)	0.460	0.999 (0.99–1.000)	0.095
ESTIMATEScore	1.000 (1.000–1.000)	0.136	0.998 (0.997–1.000)	0.101
TumorPurity	0.403 (0.125–1.306)	0.130	0.002 (0.001–1.857)	0.172
ELANE	1.311 (0.689–2.495)	0.010	1.126 (0.489–2.595)	0.018

### Relationship between ELANE and TIME in patients with GC and results of GSEA

**Figure 6:**
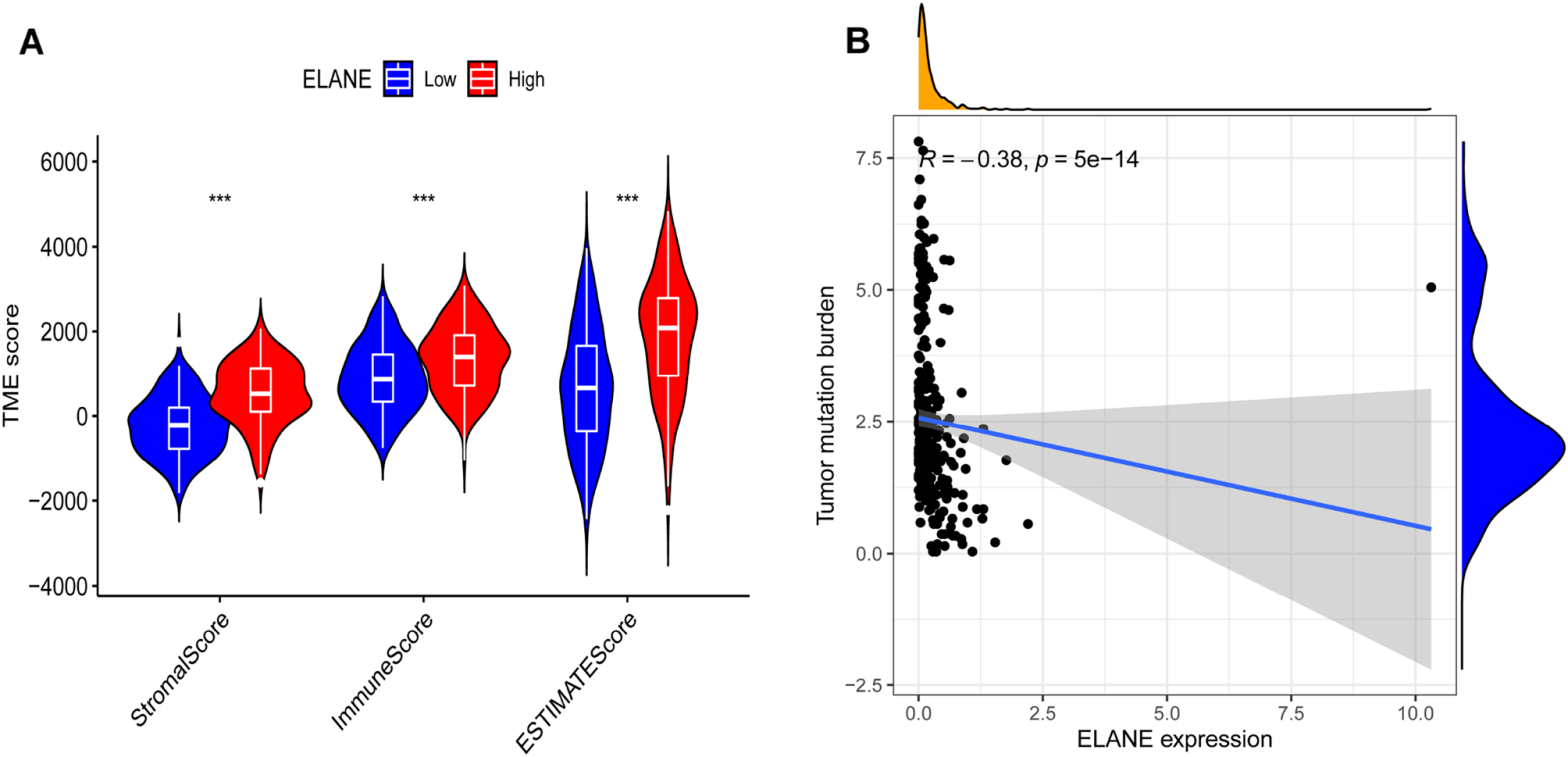
Relationship between neutrophil elastase (ELANE) expression levels and tumor immune microenvironment in patients with GC. (**A**) Relationship between ELANE expression levels and StromalScore, ImmuneScore, and ESTIMATEScore. (**B**) relationship between ELANE expression levels and tumor mutation burden.

**Figure 7:**
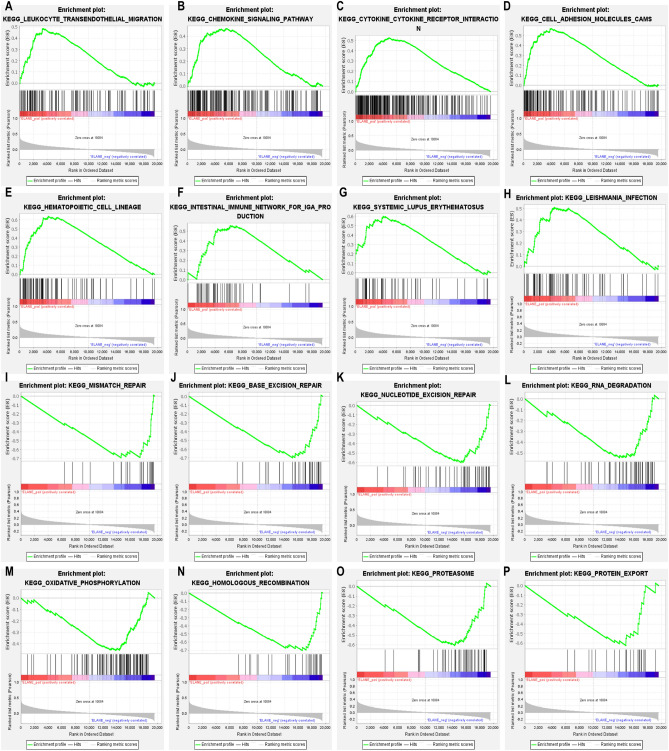
GSEA function analysis of the high ELANE expression and low ELANE expression groups. (**A**–**H**) GSEA results for samples with high ELANE expression levels. (**I**–**P**) GSEA results for samples with low ELANE expression.

This research investigated the correlation between the expression level of ELANE and TIME in patients with GC and found that the expression of ELANE was significantly correlated with the ImmuneScore, StromalScore, and ESTIMATEScore in the TIME (Fig. 6A). There was a negative correlation between the expression of ELANE and TMB (R =  − 0.38, *p* < 0.001; Fig. 6B). To explore the relevant mechanisms of ELANE and TIME, GSEA was conducted. The GSEA results indicated that the gene sets in the ELANE high-expression group were primarily involved in the signaling pathways correlated with the regulation of immune response, such as the chemokine signaling pathway, leukocyte transendothelial migration, cytokine-cytokine receptor interaction, infection, cell adhesion molecules, and autoimmune diseases (Fig. 7A–H and Table 2). The gene sets in the ELANE low-expression group were mainly involved in the signaling pathways related to metabolism, such as oxidative phosphorylation, RNA degradation, proteasomes, protein export, mismatch repair, and nucleotide excision repair (Fig. 7I–P and Table 2). The above results suggest that ELANE might be an influencing factor for the TIME to maintain the immune status.

**Table 2:** Results of the GSEA function analysis of ELANE expression levels.

c2.cp.kegg.v2022.1.Hs.symbols	Gene set name	NES	NOM *p*-val	FDR q-val
ELANE high expression	KEGG_HEMATOPOIETIC_CELL_LINEAGE	2.382	0	0
	KEGG_CELL_ADHESION_MOLECULES_CAMS	2.375	0	0
	KEGG_CYTOKINE_CYTOKINE_RECEPTOR_INTERACTION	2.180	0	0
	KEGG_LEUKOCYTE_TRANSENDOTHELIAL_MIGRATION	1.883	0	0.002
	KEGG_SYSTEMIC_LUPUS_ERYTHEMATOSUS	2.060	0	< 0.001
	KEGG_AUTOIMMUNE_THYROID_DISEASE	1.973	0	< 0.001
	KEGG_INTESTINAL_IMMUNE_NETWORK_FOR_IGA_PRODUCTION	1.883	0	0.002
	KEGG_CHEMOKINE_SIGNALING_PATHWAY	1.864	0	0.002
ELANE low expression	KEGG_BASE_EXCISION_REPAIR	− 2.712	0	0
	KEGG_MISMATCH_REPAIR	− 2.423	0	0
	KEGG_HOMOLOGOUS_RECOMBINATION	− 2.685	0	0
	KEGG_PROTEASOME	− 2.535	0	0
	KEGG_NUCLEOTIDE_EXCISION_REPAIR	− 2.505	0	0
	KEGG_RNA_DEGRADATION	− 2.354	0	0
	KEGG_OXIDATIVE_PHOSPHORYLATION	− 2.354	0	< 0.001
	KEGG_PROTEIN_EXPORT	− 2.223	0	< 0.001

### Relationship between ELANE expression levels and TIICs and ICs

**Figure 8:**
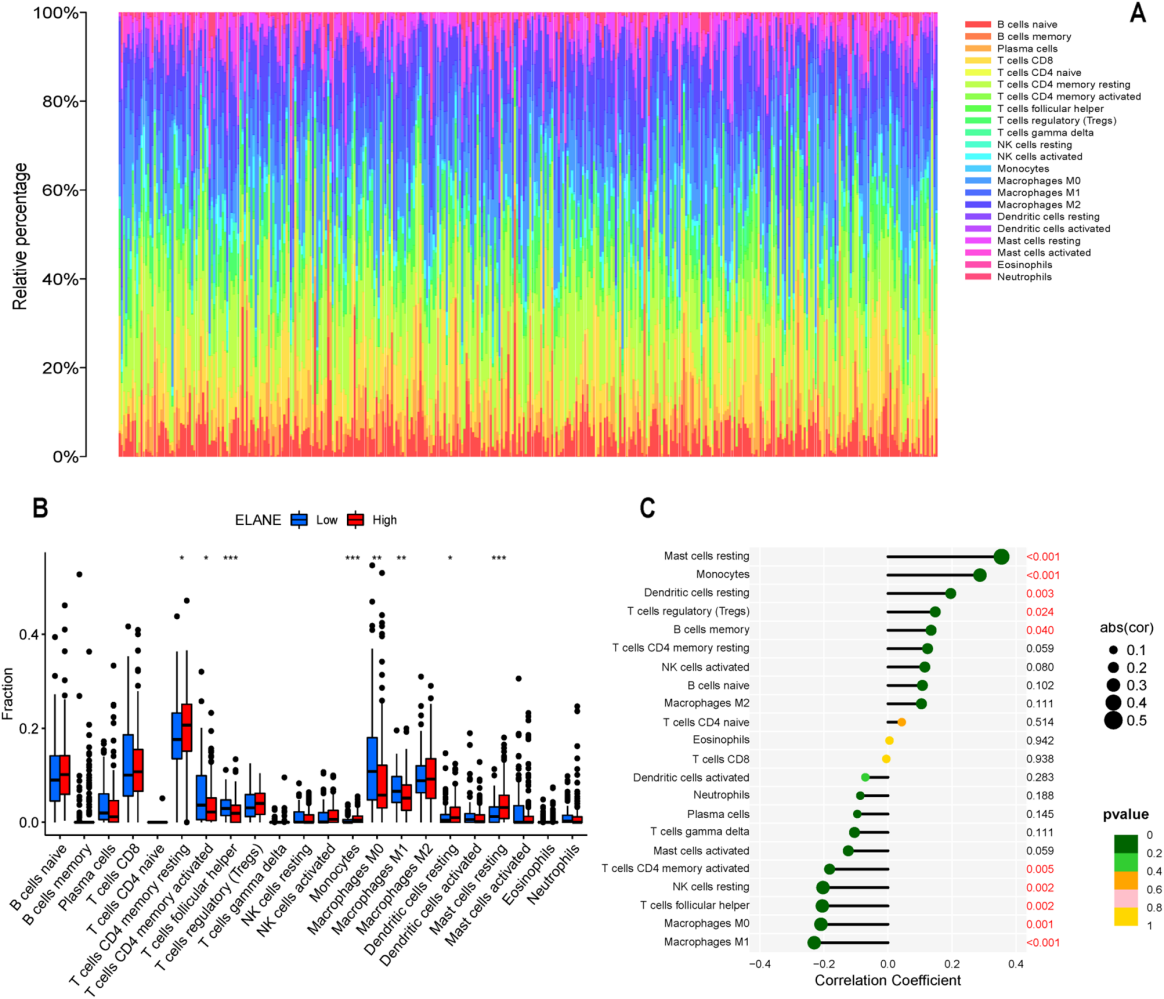
Profile of tumor immune-infiltrating cells (TIICs) in tumor samples and correlation analysis. (**A**) Bar plot showing the proportion of 22 kinds of TIICs in GC (the X-axis represents each GC sample, and the Y-axis represents the proportion of infiltration in each cell). (GC) samples. (**B**) Differences of 22 TIICs between the neutrophil elastase (ELANE) high- and low-expression group. (**C**) Correlation between ELANE expression level and TIICs.

**Figure 9:**
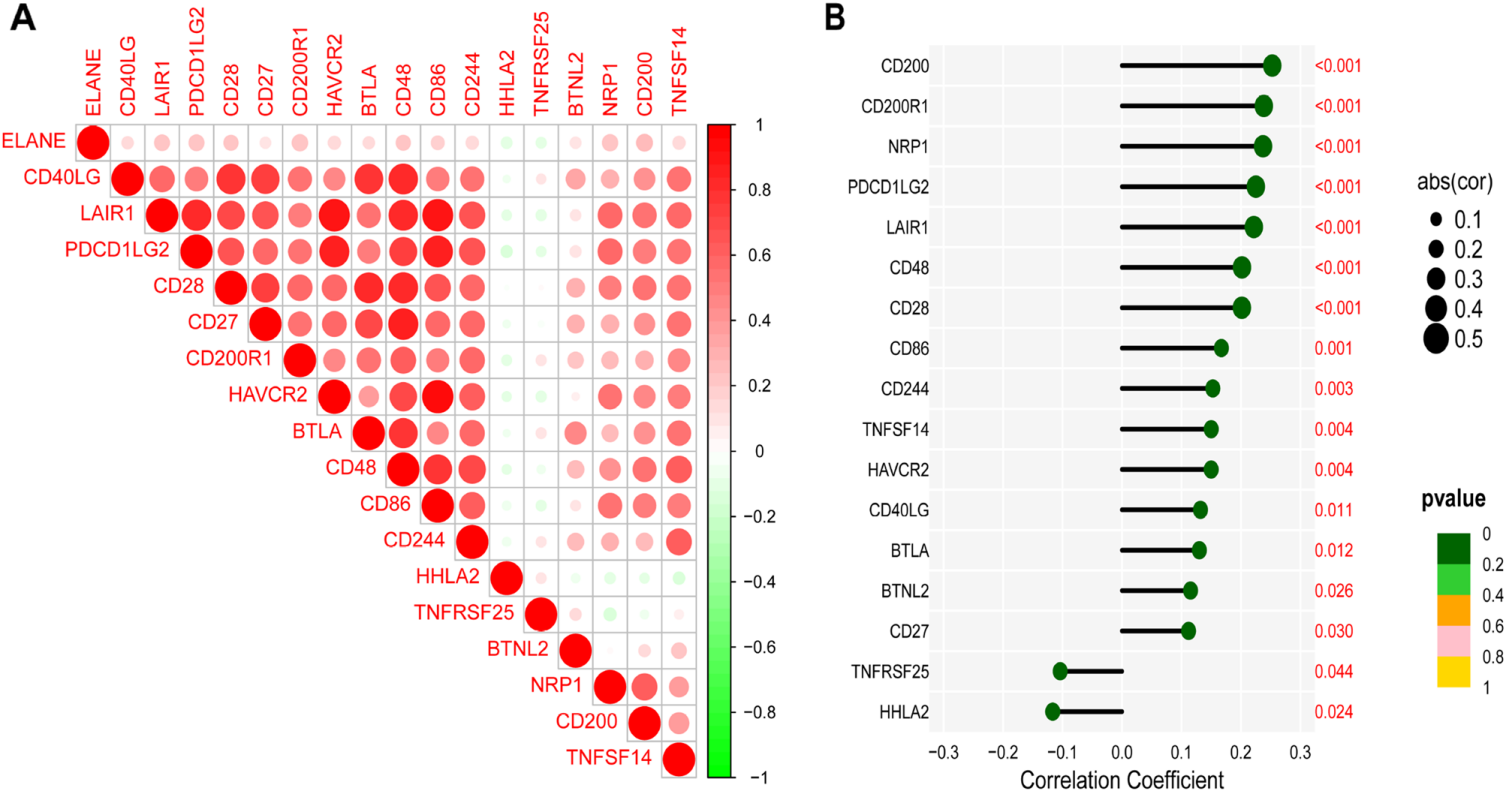
Relationship between neutrophil elastase (ELANE) expression level and immune checkpoint (IC) genes. (**A**) Seventeen kinds of ICs were closely related to the expression of ELANE. (**B**) Correlation coefficients and *p*-values between 17 kinds of ICs and ELANE expression level.

To explore and determine the relationship and mechanism between ELANE expression level and TIME in patients with GC, CIBERSORT method was applied to estimate the proportion of 22 immune cell components and quantitatively analyze the immune infiltration. The differentially expressed immune cells were screened based on the level of ELANE expression. Eight differentially expressed immune cells were screened out, including resting CD4 memory T cells, T follicular helper cells, activated CD4 memory T cells, Mono cells, M0 macrophages, M1 macrophages, resting master cells, and resting dendritic cells (Fig. 8A,B). In addition, there was a positive correlation between ELANE expression level and T-cell regulation (Tregs) (R = 0.148, *p* = 0.024), B-cell memory (R = 0.135, *p* = 0.040), resting mast cells (R = 0.355, *p* < 0.001), monocytes (R = 0.287, *p* < 0.001), and resting dendritic cells (R = 0.196, *p* = 0.003) (Fig. 8C). There was a negative correlation between ELANE expression level and the activated CD4 memory (R =  − 0.182, *p* = 0.005), resting NK cells (R =  − 0.203, *p* = 0.002), follicular helper T cells (R =  − 0.205, *p* = 0.002), M0 macrophages (R =  − 0.209, *p* = 0.001), and M1 macrophages (R =  − 0.230, *p* < 0.001) (Fig. 8C). The above results suggested that ELANE may participate in the regulation of TIICs in the TIME, and affect the TIME. To explore the clinical value of ELANE expression in immunotherapy of GC patients, we investigated the correlation between ELANE expression level and ICs gene expression level in GC. This results indicated that the expression level of ELANE was positively related to 15 kinds of IC genes (all: *p* < 0.05). The expression level of ELANE was negatively related to TNFRSF (*p* = 0.044) and HHLA2 (*p* = 0.024) (Fig. 9A,B). This finding suggests that ELANE may be a new target for immunotherapy of GC in the future.

## Discussion

At present, immunotherapy is one of the main treatments of advanced GC. However, only a few patients can benefit from immunotherapy. Immunotherapy of GC was closely related to the TIME for tumor survival. In this study, we intended to identify genes related to survival and TIME of GC patients from TCGA database. According to the results of bioinformatics analysis, ELANE might be an important biomarker for survival and TIME status of GC patients.

ELANE is a serine protease released during the degranulation of neutrophil or formation of neutrophil extracellular traps (NETs)^18^. ELANE is only expressed in mature myelomonocytes and their immature precursors. It is stored in neutrophil granules as an active protease; under normal circumstances, neutrophils express low levels of ELANE. However, during infection, neutrophils can release large quantities of ELANE to promote the growth and development of tumor^19^. Moreover, ELANE activity can promote the adhesion between pancreatic cancer cells and vascular endothelial cells by stimulating the expression of e-selectin^20^. In leukemia, ELANE gene deletion or drug inhibition can reduce the tumor mutation burden (TMB)^21^. ELANE can not only directly regulate tumor cell behavior, and is involved in the tumor-host interactions and the tumor metastasis^22,23^. Therefore, high levels of ELANE expression were considered to be risk factors in many types of tumor. Recently, Cui C et al. found that ELANE could selectively induce cell death in a variety of tumor cells by releasing CD95 death domain, meanwhile, it could also prevented tumor distant metastasis by triggering CD8 + T cell-mediated ectopic effects^24^. In addition, an elevated ELANE expression level degrades not only elastin but also other extracellular stromal proteins, including laminin and many transmembrane proteins^25,26^. ELANE also can degrade insulin receptor substrate 1 and activate the phosphatidylinositol 3 kinase-protein kinase B signaling pathway, thereby promoting the proliferation of tumor cells^27^. Therefore, the discovery of an antibody that acts on ELANE may be an effective strategy to treat tumors.

Increasing evidence has shown that a high ELANE activity is a risk factor for poor prognosis in patients with breast and lung cancer^28,29^. Through a comprehensive bioinformatics analysis of GC transcriptome data, we found that the high expression level of ELANE was closely related to the tumor progression of GC. ELANE can stimulate epithelial cells to produce inflammatory cytokines and further activate neutrophils, thereby causing an acute inflammatory response^30^. ELANE is one of the primary mediators for the formation of NETs, and NETs enriched in ELANE promote metastasis and spread by inducing epithelial-mesenchymal transition^18^. Therefore, ELANE also acts at the limit between tumor cells and the vasculature, leading to a decrease in the clearance of tumor cells inside the blood circulation^21^. In this study, the expression level of ELANE was higher in normal tissues than tumor tissues. However, the high expression of ELANE in tumor tissue was associated with poor prognosis. This may indicate that ELANE plays a negative role in the development of tumors, which may be related to the malignancy and prognosis of the tumor. ELANE is a gene that encodes enzymes, and its high expression in normal tissues may be related to the physiological function of normal cells. However, high expression in tumor tissue may be related to abnormal proliferation, invasion, metastasis, and increased treatment resistance of tumor cells. This may lead to the deterioration of the tumor and the deterioration of prognosis. However, further research is needed to determine the specific mechanism that leads to the association between high expression of ELANE in tumor tissue and poor prognosis.

In order to research the potential biological functions of ELANE, we analyzed the genes related to ELANE expression and carried out a function enrichment analysis. The KEGG pathway and GO function analyses showed that they primarily participated in immune-related processes. By examining the correlation between ELANE and the GC patients’ survival cycle, we found that the high expression of ELANE was closely related to shorter OS in GC patients. ROC result showed that the expression of ELANE had a good predictive effect on the prognosis of patients. The univariate Cox regression analysis and multivariate COX regression analyses further proved that a high expression level of ELANE is an independent risk factor for the prognosis of GC patients. These results strongly suggested that the high expression level of ELANE was significantly correlated with the poor prognosis of GC patients. This study demonstrated the value of ELANE as a clinical biomarker for GC and revealed its potential as a prognostic biomarker for GC.

The TIME consist of stromal, tumor, and immune cells, which reflect the prognosis of patients and the efficacy of tumor immunotherapy. Increasing evidence suggests that TIME affects tumor prognosis through multiple pathways; for example, stromal and immune cells can interfere with tumor signaling, thereby influencing the tumor prognosis^31^. Tumor-associated macrophages (TAMs) are one of the major stromal cells in TIME. In normal physiological environment, macrophages can differentiate into M1 macrophages and M2 macrophages. M1 can play the role of host defense and anti-tumor^32^. However, M2 plays a role in suppressing immune response by secreting anti-inflammatory cytokines^33,34^. The research of SinoSong et al. showed that ELANE can promote the polarization of M2 macrophages by downregulating PTEN, thereby accelerating the growth and development of lung cancer cells^35^. This finding suggests that ELANE might participate in the regulation of TIME macrophage polarization to promote tumor progression. Meanwhile, we found that ELANE expression was closely related to the immune score; moreover, ELANE expression was negatively correlated with TMB. To further explore the mechanism of action between ELANE and TIME, GSEA functional analysis was performed; genes in the ELANE high-expression group were primarily involved in signaling pathways correlated with the regulation of immune response, while those in the ELANE low-expression group were primarily involved in pathways correlated with metabolism, suggesting that ELANE might participate in the change of TIME from an immunodominant to a metabolically dominant state.

Based on the correlation between ELANE and TIME immune scores, we inferred that ELANE expression might affect the TIICs in the TIME. After evaluating the correlation between tumor immune infiltration, we found that M1 macrophages in ELANE low expression group were obviously increased; meanwhile, M1 macrophages can effectively kill the tumor cells^36^. We found that memory CD4 T cells and resting mast cells were also obviously increased in the ELANE high-expression group; however, the tumor infiltration of these cells was related to poor prognosis in tumor patients^37^. Simultaneously, ELANE expression was positively related to Tregs; however, the higher degree of Treg infiltration in tumors was significantly correlated with the poor prognosis^38^. These results indicated that high expression of ELANE can stimulate the infiltration of immune cells associated with poor prognosis, which further illustrated the correlation between ELANE expression and the GC patients’ prognosis. The above results also suggested that, with the progression of GC, the expression status of ELANE changed from being upregulated to being downregulated, and TIME also changed from immune dominant to metabolically dominant, leading to the reduction the levels of antitumor TICs. The results of this study also showed that ELANE is significantly correlated with a variety of immune checkpoint genes, thus suggesting that ELANE may be an effective target of immunotherapy in the future.

In conclusion, ELANE might be a potential prognostic biomarker of GC, which can be used as one of the targets of TIME immunotherapy and provide a reference for GC research. Of course, this research has the following limitations. First, it was a retrospective study based on a single database; second, the accuracy of ELANE in predicting the GC patients’ prognosis requires further verification in the actual clinical practice. In addition, the mechanism of ELANE's influence on GC progression and tumor immune invasion needs to be further clarified.

## Conclusion

A high ELANE expression level is an independent risk factor for the GC patients’ prognosis. ELANE might be a potential biomarker that can predict the survival and prognosis of patients with GC. ELANE might affect the GC patients’ prognosis by affecting the tumor immune cell infiltration in the TIME. It might be one of the factors affecting the TIME to maintain an immune status.

## Data Availability

The data used in this study are freely available in The Cancer Genome Atlas database (https://portal.gdc.cancer.gov). The analytical data, original figures, and other information related to this study can be obtained from the corresponding author.

## Abbreviations

GC: Gastric cancer
TIME: Tumor immune microenvironment
ELANE: Neutrophil elastase
TCGA: The cancer genome atlas
DGEs: Differentially expressed genes
FDR: False discovery rate
GO: Gene ontology
KEGG: Kyoto encyclopedia of genes and genomes
PPI: Protein–protein interaction network
K–M: Kaplan–Meier
GSEA: Gene set enrichment analysis
AUC: Area under the curve
TIICs: Tumor immune-infiltrating cells
NETs: Neutrophil extracellular traps
TMB: Tumor mutation burden
